# Engineering vascular grafts from decellularized plants: Advances and challenges

**DOI:** 10.14670/HH-18-934

**Published:** 2025-05-08

**Authors:** Nick Merna

**Affiliations:** Fred DeMatteis School of Engineering and Applied Science, Hofstra University, New York, USA

**Keywords:** Vascular grafts, Tissue engineering, Extracellular matrix, Plant scaffolds, Decellularization

## Abstract

Small-caliber vascular grafts (<6 mm diameter) are critical for coronary and peripheral bypass surgeries, yet developing functional substitutes remains challenging. Autologous vessels are ideal but often unavailable or of poor quality. Synthetic grafts, such as expanded polytetrafluoroethylene (ePTFE) and Dacron, have high failure rates in small diameters due to thrombosis, intimal hyperplasia, and compliance mismatch. Tissue-engineered vascular grafts (TEVGs) aim to overcome these issues by providing a biocompatible scaffold with an endothelial lining.

Decellularized plant tissues have recently gained attention as natural scaffolds for TEVGs due to their structural similarity to human vasculature. Leaves and stems provide an extracellular matrix (ECM) primarily composed of cellulose, which is biocompatible, porous, and non-thrombogenic. These scaffolds are cost-effective, scalable, and ethically uncontroversial. Decellularized parsley stems or leatherleaf leaves, for instance, can be recellularized with endothelial and smooth muscle cells (SMCs) to create small-diameter grafts that support endothelialization and withstand physiological pressures.

Perfusion bioreactors further enhance the functionality of plant-based grafts by simulating physiological conditions. Pulsatile flow and pressure stimulate endothelial cell alignment, reducing thrombogenicity, while mechanical stimulation promotes SMC maturation and ECM deposition, improving graft strength and compliance.

This review summarizes recent advances in plant-based vascular grafts and perfusion bioreactor conditioning, compares their performance to conventional grafts, and highlights remaining challenges. Decellularized plant scaffolds, with their inherent vascular architecture and biocompatibility, show promise as natural templates for small-caliber vascular grafts. However, further research is needed to address key challenges such as standardization, mechanical optimization, and long-term *in vivo* validation to facilitate their clinical application.

## Introduction

Small-diameter vascular grafts remain a significant challenge in tissue engineering due to the high failure rates of existing options. Cardiovascular disease is the leading cause of mortality worldwide, accounting for 19 million deaths annually (Tsao et al., 2023). Autologous vessels, including the saphenous vein and internal mammary artery, are preferred for bypass grafts. However, many patients lack suitable donor vessels because of prior harvest or disease (Chew et al., 2002; Sabik 3rd, 2011).

Although synthetic grafts like ePTFE and Dacron perform well in large-caliber arteries, they fail at diameters below 6 mm due to thrombosis, compliance mismatch, and intimal hyperplasia (Crombez et al., 2005; Pashneh-Tala et al., 2016; Obiweluozor et al., 2020). Thrombosis results from the absence of an endothelial monolayer, leading to platelet adhesion and clotting (Lu and Sipehia, 2001). Efforts to improve the endothelialization of synthetic grafts have included immobilization of vascular endothelial growth factor (VEGF) onto PTFE to enhance endothelial cell adhesion and migration (Crombez et al., 2005). Compliance mismatch at anastomotic sites also leads to intimal hyperplasia, the abnormal thickening of the vessel’s inner lining, where SMC proliferation and ECM deposition contribute to graft stenosis (Ballyk et al., 1998).

TEVGs aim to overcome these limitations by providing biocompatible scaffolds that promote endothelialization while matching the mechanical properties of native vessels. Biologically-derived materials, such as decellularized small intestine submucosa and umbilical arteries, have been explored as scaffolds that retain native ECM structure (Badylak et al., 2009; Gui et al., 2009; Palmosi et al., 2022; Wong et al., 2022). Decellularized human umbilical arteries, for instance, have shown favorable mechanical properties and endothelialization potential. CHAPS and SDS detergents effectively remove cells while preserving ECM integrity, but challenges remain, including reduced burst pressure and limited long-term validation (Wong et al., 2022). While some decellularized animal grafts have shown promise, they face limitations, including limited supply, high processing costs, and potential xenogeneic risks (Huerta et al., 2016). Consequently, there is strong motivation to identify alternative biomaterials that are readily available, biocompatible, and conducive to endothelialization. Since hypertension-related deaths increased by 34.2% from 1999 to 2019, and diabetes continues to rise as a leading cardiovascular risk factor, improved vascular grafts that mitigate thrombogenicity and failure are crucial (Tsao et al., 2023).

Decellularized plant tissues have recently emerged as a promising solution, offering unique advantages over synthetic and animal-derived grafts (Gershlak et al., 2017; Modulevsky, 2021). Plants possess extensive vascular networks and diverse architectures that can mimic human tissue structures (Gershlak et al., 2017; Zhu et al., 2021). Notably, plant cell walls are composed mainly of cellulose, a natural polymer known for its biocompatibility, low thrombogenicity, and controlled permeability (Miyamoto et al., 1989; Caffall and Mohnen, 2009; Fontana et al., 2017; Mallik et al., 2019; Gorbenko et al., 2024). Unlike animal ECM, cellulose lacks proteins that trigger coagulation, reducing thrombogenic risk while supporting human cell attachment and growth. Additionally, plant scaffolds are inexpensive, free from ethical concerns, and can be produced in abundance (Zhu et al., 2021). By decellularizing leaves, stems, or woody tissues, one can obtain scaffolds with pre-existing microchannels, high porosity, and tunable mechanical properties, making them particularly attractive for vascular tissue engineering (Fernandes et al., 2011; Adamski et al., 2018; Harris et al., 2021). However, variability in decellularization protocols and material sources significantly impacts scaffold properties, particularly mechanical strength and cellular compatibility, necessitating further standardization (Imeidopf et al., 2025). Despite these challenges, recent optimizations in plant-derived scaffold processing have demonstrated their potential as viable templates for small-caliber vascular grafts, warranting further investigation for clinical translation.

Functional vascular grafts require more than cell seeding; they must undergo dynamic conditioning in a perfusion bioreactor, a system that mimics blood flow to enhance cellular integration and mechanical strength (Mitchell et al., 2023). Plant-derived scaffolds can be successfully seeded with endothelial cells and SMCs to recreate the layered architecture of native blood vessels, where the endothelium provides a non-thrombogenic surface, and SMCs regulate vascular tone and structural integrity. Exposing grafts to fluid shear stress and cyclic pressure induces endothelial cell alignment and robust monolayer formation while also stimulating the contractile phenotype of SMCs (Alkazemi et al., 2023). This dynamic conditioning reduces thrombogenicity and strengthens the tissue prior to implantation (Gorbenko et al., 2024).

Plant-derived scaffolds have been investigated for bone, muscle, and cardiac tissue engineering, providing valuable insights for vascular graft development. The structural properties of plant-derived cellulose that make it suitable for tissue scaffolds, such as porosity, biocompatibility, and adaptability, are being explored for diverse biomedical applications. Examining these applications provides insight into potential modifications that could enhance vascular graft functionality, such as improved mechanical properties, biodegradability, and cell integration.

Plant-derived scaffolds with perfusion bioreactor conditioning offer a promising approach to overcoming current challenges in small-diameter vascular graft development. While plant-derived scaffolds have been explored for applications in wound healing and bone regeneration, their potential for vascular grafts has only been recently explored. Since 2023, studies have demonstrated that plant scaffolds can support endothelialization, withstand physiological pressures, and exhibit favorable mechanical properties, highlighting the need for a comprehensive review of these advances (Cevik and Dikici, 2023; Gorbenko et al., 2023). Previous reviews have summarized plant decellularization in general, but none have specifically focused on their role as vascular grafts or integrated the latest advances in bioreactor conditioning and comparative performance. This review examines the latest research on plant-derived vascular grafts, focusing on (i) methods for decellularizing different plant sources, (ii) recent progress in their use for vascular graft applications, (iii) the role of perfusion bioreactor conditioning in improving graft performance, (iv) broader applications of plant ECM in tissue engineering, and (v) remaining challenges and future directions for clinical translation.

## Plant-based decellularized scaffolds in tissue engineering

**Fig. 1.**
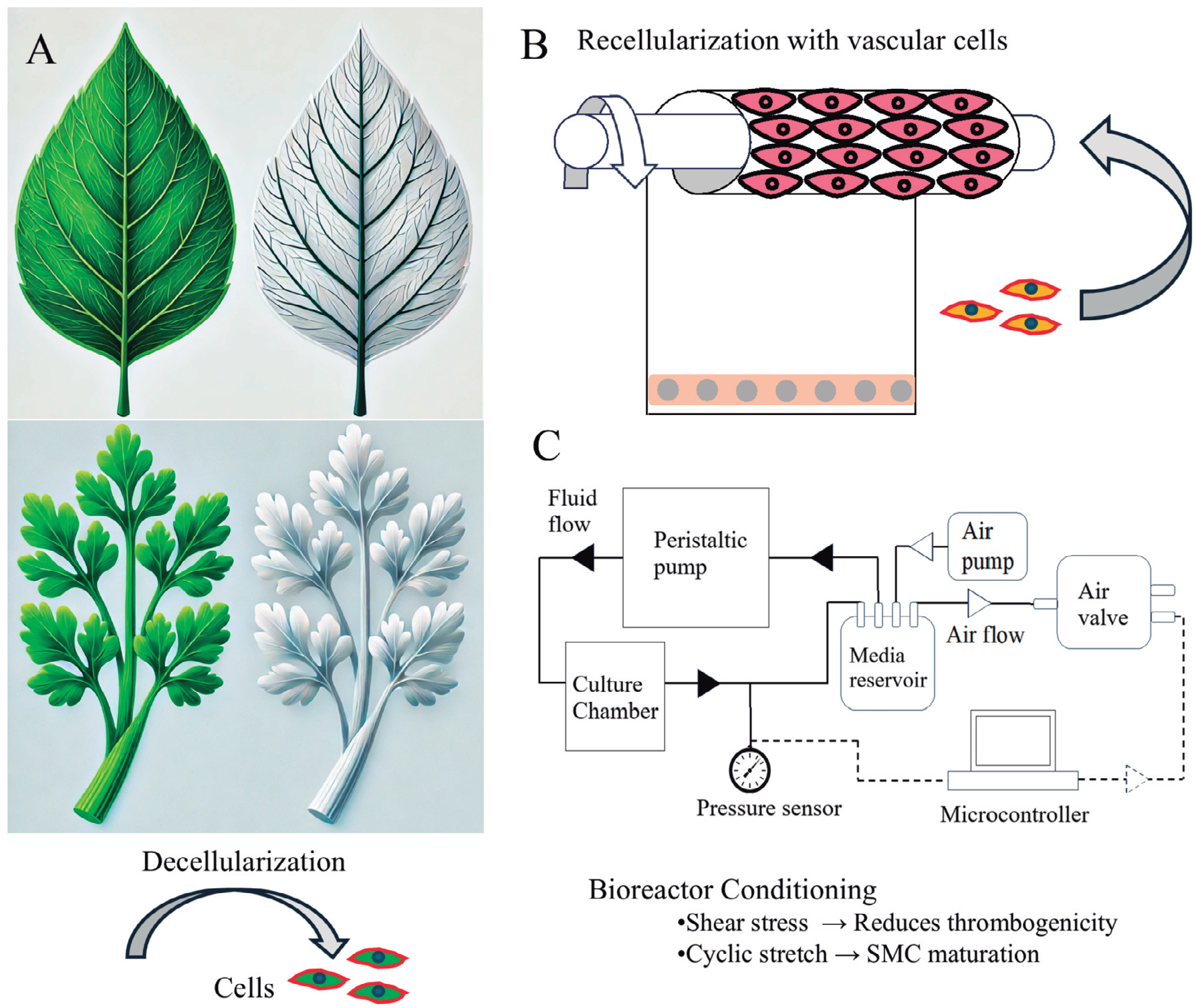
Development of plant-derived vascular grafts. Decellularization of plant leaves or stems with SDS, Triton X-100, and clearing solution **(A)**, followed by recellularization of scaffolds with vascular cells **(B)**, and bioreactor conditioning of the tissue constructs **(C)**.

In general, the fabrication of plant-based vascular grafts follows three key phases: decellularization, recellularization, and bioreactor conditioning (Fig. 1). The goal is to remove immunogenic cellular components while preserving the 3D cellulose network that provides mechanical stability. In the recellularization phase, endothelial and SMCs are seeded onto the scaffold to restore vascular functionality, promote thromboresistance, and enhance mechanical integrity. This process is further refined through bioreactor conditioning, where exposure to physiological flow and cyclic strain strengthens cell adhesion, induces native-like alignment, and stimulates ECM deposition, ultimately improving graft performance before implantation.

### Decellularization principles

Plant decellularization involves removing all cellular components from plant tissue while preserving the structural matrix of cellulose and other polysaccharides, such as hemicellulose, pectin, and lignin (Zablackis et al., 1995; Caffall and Mohnen, 2009). The primary goal is to eliminate plant DNA and antigens that could elicit immune responses while maintaining the scaffold’s architecture and mechanical properties. Effective decellularization yields a whitish and translucent scaffold, indicating the absence of pigmented cells, while retaining an intricate network of channels that mimic capillary structures (Gershlak et al., 2017). These channels can be perfused with blood or culture media, simulating the function of blood vessels. Notably, cellulose is generally recognized as bio-inert in mammals, demonstrating excellent biocompatibility *in vivo*. Modulevsky et al. implanted decellularized apple scaffolds subcutaneously in mice, observing only a mild foreign body reaction that resolved over time, with the cellulose scaffold integrating and supporting new tissue formation (Modulevsky et al., 2016). This suggests that properly decellularized plant matrices can serve as safe biomaterials for tissue engineering.

Various protocols have been developed to decellularize plant tissues, often adapting techniques from animal tissue decellularization. Key methods include:

#### Detergent-based decellularization

Ionic or non-ionic detergents are perfused or agitated through the plant vasculature to lyse cells. Sodium dodecyl sulfate (SDS) and Triton X-100 are widely used (Fontana et al., 2017), with SDS effectively solubilizing cell membranes and removing proteins, while Triton X-100 can help remove cells while preserving ECM structure. Some protocols employ a combination, using SDS for primary decellularization and Triton X-100 washes to clear residual debris. For instance, spinach, parsley, and leatherleaf viburnum leaves have been treated with 2% SDS, followed by clearing with Triton X-100 and bleach solution, yielding transparent decellularized leaf scaffolds (Gorbenko et al., 2023). These techniques have demonstrated >90% DNA removal while maintaining vasculature architecture. Recent findings indicate that shorter clearing times achieve high DNA removal (>95%) while preserving mechanical properties, whereas prolonged treatments can weaken scaffold integrity (Imeidopf et al., 2025).

Alternative protocols using Tergitol and EGTA have been explored; however, these methods resulted in incomplete decellularization, leaving behind residual cellular material that compromised cell seeding efficiency (Gorbenko et al., 2023; Imeidopf et al., 2025). Tergitol is a milder, non-ionic surfactant, making it less efficient at removing nuclear material and embedded cells. EGTA, a calcium chelator commonly used for disrupting cell adhesion, did not sufficiently remove embedded cells. In contrast, SDS is an ionic detergent that aggressively disrupts lipid membranes and solubilizes intracellular components, effectively removing cellular material from plant scaffolds. Triton X-100, a non-ionic surfactant, primarily works by disrupting lipid-lipid and lipid-protein interactions, while bleach (sodium hypochlorite) acts as a strong oxidizing agent, aiding in the further removal of cellular remnants.

#### Enzymatic treatments

Specific enzymes target cellular components without damaging the ECM. Deoxyribonuclease (DNase) digests residual DNA after detergent treatment, ensuring complete removal of nucleic material (Moffat et al., 2022; Yazdi et al., 2024). Some studies suggest that DNase alone (1 mg/mL) can effectively decellularize certain plant tissue while preserving proteins (Phan et al., 2020). Additionally, enzymatic cocktails containing pectinase or cellulase may further aid in breaking down cellular material, though excessive use risks damaging the cellulose scaffold. Alternative protocols using Trypsin, a proteolytic enzyme, have been explored; however, these methods resulted in incomplete decellularization, leaving behind residual cellular material (Imeidopf et al., 2025).

#### Supercritical carbon dioxide (scCO_2_)

A novel, non-toxic decellularization technique, scCO_2_ uses CO_2_ at high pressure and moderate temperature to penetrate tissues (Harris et al., 2021). The supercritical fluid can infiltrate plant cell walls and, upon rapid depressurization, disrupt cells and extract lipids and proteins. When combined with peracetic acid, scCO_2_ efficiently removes plant cells while preserving the scaffold’s mechanical properties. This method has been successfully used to decellularize spinach leaves, yielding scaffolds comparable to detergent-based methods but with fewer alterations in mechanical integrity.

#### Physical methods

Freeze–thaw cycles help disrupt plant cell membranes by ice crystal formation (Thippan et al., 2019), while agitation and sonication facilitate reagent penetration. Simply soaking tissue in water to allow natural degradation of soft plant matter (an old technique from the 18th century) has even been used for very delicate tissues, though this is time-consuming. In most cases, physical methods are used in combination with chemical or enzymatic treatments to achieve complete decellularization.

Regardless of the method, a critical quality metric is the residual DNA content, which should be reduced to <50 ng per mg scaffold (a standard threshold for biomaterials safety). Histological staining (Hematoxylin & Eosin) and nuclear staining (DAPI or Hoechst) confirm cell removal, while DNA analysis ensures effective decellularization. For example, in decellularized parsley stem scaffolds, >95% DNA reduction has been reported, with no intact plant cells detected (Cevik and Dikici, 2023). Additionally, thorough post-treatment washing is essential to remove residual detergents and reagents. For example, residual SDS can be cytotoxic to cells and hinder recellularization if not fully rinsed away. Achieving complete decellularization and post-treatment washing is essential to minimize immune responses and optimize biocompatibility.

### Structural properties

The appeal of plant scaffolds lies in their diverse structures, which can be tailored to different tissue engineering applications. Comparisons between synthetic, biodegradable, and natural vascular graft materials indicate that scaffold stiffness, compliance, and degradation rates significantly impact long-term function (Obiweluozor et al., 2020). Leaves have a highly vascularized network of fine capillary-like veins embedded in a soft parenchymal matrix, while stems provide cylindrical geometries with greater axial strength, making them ideal for vascular graft applications (Jauneau et al., 2020). Decellularized spinach leaves, for example, maintain an open channel network suitable for perfusing fluids, suggesting potential uses for cardiac or hepatic tissue engineering (Gershlak et al., 2017).

For vascular applications, parsley and bamboo stems offer naturally hollow structures with sufficient mechanical strength to support suturing and pulsatile flow (Lacombe et al., 2020). Studies indicate that parsley stem grafts are soft yet resilient, with burst pressures comparable to native arteries. However, plant scaffolds lack elastin, requiring modifications such as elastomer embedding or cross-linking to match arterial compliance. Decellularized plant scaffolds provide a versatile and sustainable approach for vascular tissue engineering. Their naturally porous architecture, high tensile strength, and flexibility make them well-suited for mimicking native blood vessels. Combined with their biocompatibility and non-thrombogenic properties, they present a compelling alternative to synthetic and animal-derived grafts.

## Comparison of plant sources for vascular grafts

Various plant tissues have been explored as scaffolds for small-diameter vascular grafts (<6 mm), leveraging their natural vascular networks and cellulose-based biocompatibility. Studies have evaluated different plant sources for mechanical integrity, recellularization potential, and endothelialization (Gorbenko et al., 2023). Both leaves and stems offer unique advantages in plant-based vascular graft engineering. Leaves such as spinach and leatherleaf viburnum provide a dense capillary-like network, while stems, such as parsley, offer naturally tubular structures that better replicate native blood vessels.

### Leaf-based scaffolds

Decellularized spinach, leatherleaf viburnum, and parsley have been used to engineer small-caliber vascular grafts. Gorbenko et al. demonstrated that decellularized leatherleaf viburnum, reinforced with cross-linked gelatin, produced small-diameter grafts with adequate tensile strength, burst pressure, and endothelial cell attachment. The native vein structure of leaves provides a branching network ideal for microvascular applications, although their relatively flat geometry requires rolling into tubular forms for vascular graft fabrication. Spinach, a widely studied plant model, maintains an open channel network post-decellularization, yet its delicate structure poses challenges to long-term mechanical stability.

### Stem-based scaffolds

More recently, parsley stems have emerged as a viable alternative for vascular graft engineering (Cevik and Dikici, 2023). Their natural hollow tubes reduce the need for additional fabrication steps such as rolling or molding. Their decellularization yields soft yet mechanically resilient tubular scaffolds that sustain endothelial cell adhesion and physiological pressures. Unlike leaves, stem-based scaffolds offer a preformed luminal structure with minimal compliance mismatch, which is a common issue in synthetic grafts.

## Applications for vascular tissue engineering

Decellularized plant scaffolds have recently shown promise for the fabrication of small-caliber vascular grafts. Researchers have explored various plant sources, from delicate leaves to herbaceous stems, as the structural backbone for vascular graft constructs. The standard fabrication process involves decellularizing plant tissue, shaping it into a vessel-like form if necessary, and recellularization with vascular cells to create a functional blood vessel substitute.

### Fabrication of plant-derived vascular grafts

One approach is to use naturally tubular plant structures. Cevik and Dikici recently demonstrated this using parsley stems, which contain a narrow central cavity that remains intact after decellularization (Cevik and Dikici, 2023). SDS-based decellularization effectively removed cellular material while preserving the scaffold’s flexibility, eliminating the need for additional fabrication steps.

**Fig. 2.**
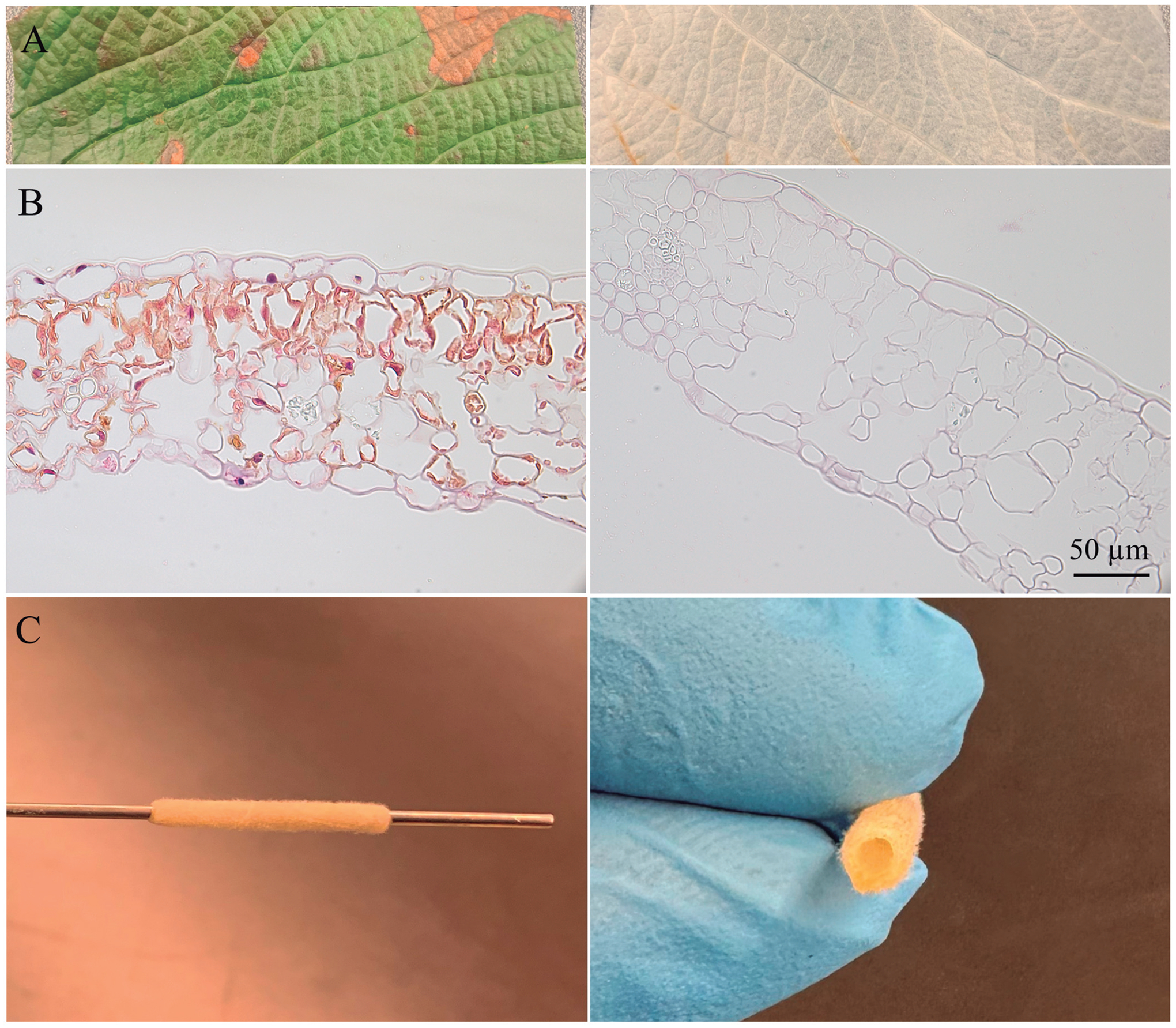
Leatherleaf viburnum scaffolds before and after decellularization with SDS. **A.** Macroscopic images. **B.** H&E staining confirms cell removal. **C.** Rolled graft for vascular applications.

Another approach involves modifying flat plant-derived scaffolds to create tubular grafts. Gorbenko et al. engineered small-diameter grafts by rolling leatherleaf viburnum leaves into multilayered tubes, reinforcing them with cross-linked gelatin to improve mechanical strength and durability (Fig. 2). These leaf-derived grafts, with lumen diameters of 1–2 mm, were biocompatible, non-thrombogenic, and mechanically stable, making them potential candidates for coronary or femoral artery replacements. Increased cross-linking improves mechanical integrity while reducing swelling and degradation rates (Bigi et al., 2001; Deiber et al., 2009; Suzuki and Ikada, 2013).

### Recellularization with vascular cells

To establish a non-thrombogenic surface, plant-based scaffolds must be seeded with endothelial cells. While cellulose is not inherently coagulative, it accumulates platelets if exposed to blood without an endothelium. In the parsley stem graft study, human endothelial cells were cultured on the lumen of the decellularized stems, where they adhered and formed a confluent pseudo-endothelium layer. Histological analysis confirmed cell attachment and the development of cobblestone morphology. Similarly, Gorbenko et al. seeded rat aortic endothelial cells onto decellularized leaf scaffolds (both flat and tubular), observing viable and proliferative cells after 14 days, achieving comparable densities to conventional collagen scaffolds (Gorbenko et al., 2023). This suggests that plant cellulose provides a suitable surface for endothelial cell adhesion and proliferation.

Beyond endothelialization, SMCs play a key role in replicating the tunica media of native vessels. In a follow-up study, Gorbenko et al. seeded rat aortic endothelial and SMCs onto decellularized leatherleaf viburnum grafts (Gorbenko et al., 2024). SMCs were seeded onto 2D sheets of decellularized leaves before fabrication of the tubular structure, and then endothelial cells were perfused into the lumen of the graft. Under dynamic culture conditions, SMCs adopted a contractile phenotype and deposited ECM, suggesting the early stages of functional vessel formation. While a healthy endothelial lining is crucial for thromboresistance, SMCs contribute to long-term stability and vasoactive function, making their incorporation into plant-based grafts a priority for translational applications. With viable endothelial and SMC colonization achieved on plant scaffolds, the next critical consideration is how these constructs perform relative to conventional grafts and under physiological conditions.

### Comparative performance and biological evaluations of plant-derived vascular grafts

Early evaluations indicate that plant-derived vascular grafts can match or exceed the performance of certain synthetic and decellularized animal grafts *in vitro* (Table 1). A key advantage of cellulose-based scaffolds is their modifiability. Functionalization with bioactive molecules, such as heparin for anticoagulation, can further enhance hemocompatibility (Ishihara and Nakabayashi, 1995; Svensson et al., 2021). Mechanical evaluations confirm that plant-based grafts meet essential structural criteria.

Decellularized parsley stems demonstrated burst pressures exceeding 400 mmHg, well above physiological blood pressure while maintaining compliance similar to native vessels (Cevik and Dikici, 2023). Leatherleaf viburnum leaf-gelatin grafts had burst pressures of 2000 mmHg and exhibited tensile strength comparable to native coronary arteries (Gorbenko et al., 2023). Importantly, suture retention tests confirmed that plant-derived grafts could withstand surgical handling, an essential benchmark for clinical translation (Gorbenko et al., 2024).

By comparison, many synthetic small-diameter grafts exhibit high stiffness, leading to compliance mismatch and intimal hyperplasia at anastomotic sites (Sarkar et al., 2006). Compliance mismatch increases suture-line stress, particularly in end-to-side anastomoses, promoting smooth muscle proliferation and ECM deposition, ultimately causing graft failure (Ballyk et al., 1998). Plant scaffolds, particularly soft and compliant options like parsley stems, more closely match arterial compliance, potentially mitigating these issues.

In static and dynamic perfusion tests, SDS-decellularized leatherleaf viburnum scaffolds demonstrated minimal leakage, with an average permeability of 0.24 ± 0.05 mL/cm^2^/min at 120 mmHg, a level comparable to commercially available collagen-coated Dacron grafts (Imeidopf et al., 2025). This controlled permeability suggests that plant-derived scaffolds can provide adequate barrier function to prevent excessive leakage while still allowing for nutrient diffusion and cellular infiltration, essential for endothelialization and host tissue integration. Unlike synthetic grafts, which may require preclotting to reduce excessive porosity, plant-based grafts exhibit tunable permeability based on decellularization parameters, making them promising candidates for further optimization in vascular applications.

The ability of plant-derived grafts to maintain patency and resist thrombosis under physiological conditions is crucial for their clinical potential. Endothelialized plant-based grafts have demonstrated significantly reduced platelet adhesion and fibrin deposition compared to unseeded scaffolds (Gorbenko et al., 2024). In dynamic blood-contacting assays, endothelialized leaf-gelatin grafts exhibited virtually no thrombus formation after 30 min of perfusion with whole blood, whereas unseeded grafts developed thrombi when perfused with whole blood. These findings underscore the importance of pre-seeding plant scaffolds with endothelial cells to minimize thrombosis risk. Notably, plant cellulose itself may provide additional antithrombogenic properties. Unlike collagen-rich animal-derived scaffolds, cellulose lacks the proteins that trigger coagulation, potentially creating a benign blood-contacting surface when endothelialized (Modulevsky et al., 2016).

Preliminary *in vivo* studies suggest promising biocompatibility. Subcutaneous implantation of plant-derived cellulose scaffolds resulted in minimal fibrous capsule formation and low inflammatory responses (Modulevsky et al., 2016). Host cell infiltration and ECM deposition were observed within four weeks. In a chorioallantoic membrane (CAM) model, decellularized spinach leaf scaffolds supported host blood vessel infiltration and sustained perfusion without thrombotic occlusion (Dikici et al., 2019). This early evidence indicates that plant-derived scaffolds can integrate with host vasculature and support blood flow. However, full-scale vascular graft implantation in animal models has not yet been widely reported. Ongoing studies aim to implant leatherleaf viburnum vascular grafts as aortic interpositions in a rat model, evaluating patency and remodeling over several months. Since plant scaffolds lack immunogenic proteins found in decellularized animal ECM, they may evade immune rejection when endothelialized with autologous cells. Long-term studies will be needed to confirm these hypotheses and assess graft remodeling, endothelial retention, and functional durability under arterial flow conditions. While the early results are encouraging, further enhancement of graft performance, particularly via biomechanical conditioning, will be necessary to meet the demands of arterial implantation.

## Perfusion bioreactors for graft conditioning

Mechanical conditioning plays a critical role in the maturation of TEVGs, replicating physiological forces to enhance graft function before implantation. *In vivo*, blood vessels experience continuous shear stress from blood flow and cyclic strain from pulsatile pressure, both of which regulate endothelial and SMC function. Perfusion bioreactors aim to simulate these biomechanical conditions, promoting endothelialization, ECM deposition, and structural remodeling in engineered grafts (Bilodeau et al., 2005).

### Bioreactor design and function

A typical perfusion bioreactor system consists of a flow loop that pumps culture medium through the graft lumen, often using a peristaltic pump to generate pulsatile flow (Fig. 1C). To better replicate physiological conditions, pressure chambers or compliance elements are incorporated to simulate arterial pressure waveforms (e.g., 120/80 mmHg). In a recent study, Gorbenko et al. developed a closed-loop perfusion bioreactor for conditioning plant-derived vascular grafts (Gorbenko et al., 2024). This system precisely controlled flow rate and pressure in real-time, modulating pressure between 50–120 mmHg at 525 beats per minute (8.75 Hz) to mimic small-animal circulation. The grafts were mounted inside a sterile bioreactor chamber, where endothelial cells were exposed to laminar shear stress, and the vessel wall experienced cyclic circumferential stretch. These biomechanical cues are essential for vascular graft maturation, ensuring that cells align, adhere, and secrete ECM components necessary for long-term function.

### Mechanical stimulation benefits

Within 24 hours of exposure to flow, endothelial cells sense shear stress (10–20 dyn/cm^2^) and align in the direction of flow, mimicking native arterial endothelium (Merna et al., 2018). This alignment enhances endothelial adhesion, reducing cell detachment upon implantation and lowering thrombogenicity (Miyata et al., 1991). Shear stress also regulates mechanotransduction pathways, influenced by substrate stiffness and ECM composition, that govern endothelial morphology and function (Bacci et al., 2021).

At the molecular level, shear stress upregulates key endothelial genes, including nitric oxide synthase and Krüppel-like factors, which maintain the cells in an antithrombogenic and anti-inflammatory state (Hamik et al., 2007; Nayak et al., 2011). Studies show that laminar shear suppresses VCAM-1 expression, reducing leukocyte adhesion and promoting nitric oxide production, critical for vasodilation and platelet inhibition (Niebauer and Cooke, 1996; Pan, 2009). This conditioning helps create a stable, non-thrombogenic luminal surface, essential for long-term graft function.

Simultaneously, cyclic strain (5–10% radial stretch at 1–2 Hz) influences SMCs within the graft wall, promoting their transition to a contractile phenotype characterized by proteins such as smooth muscle myosin heavy chain and calponin (Reusch et al., 1996). In static culture, SMCs remain in a synthetic, proliferative state, leading to excessive ECM deposition and intimal hyperplasia. However, under cyclic strain, SMCs mature into contractile cells that better regulate vessel tone and function (Eoh et al., 2017).

Beyond cellular alignment and phenotype, dynamic conditioning enhances ECM remodeling. The cyclic strain increases elastin synthesis, improving graft elasticity while stimulating collagen deposition and strengthening the vessel wall (Cummings et al., 2012). These biomechanical cues drive tissue remodeling, improving burst pressure and suture retention, critical parameters for surgical implantation and long-term durability.

### Effects on plant-based grafts

The application of perfusion bioreactor conditioning to plant-derived vascular grafts was first demonstrated by Gorbenko et al. (2024). This study subjected decellularized leatherleaf viburnum grafts seeded with endothelial cells and SMCs to pulsatile flow for three weeks. The results showed that perfusion conditioning significantly increased endothelial cell density on the graft lumen compared to static culture. Endothelial cells exposed to shear stress formed more confluent monolayers, which improved adhesion and reduced the likelihood of detachment upon implantation. Conditioned grafts also exhibited less fibrin and platelet deposition when exposed to whole blood, suggesting enhanced thromboresistance.

Pulsatile flow and pressure helped maintain SMC viability within the graft wall by improving nutrient diffusion and oxygen transport. In static culture, cells deep within the scaffold often experience hypoxia and nutrient deprivation, limiting their survival and function. By promoting convective transport, perfusion bioreactors ensured that SMCs remained viable and capable of depositing ECM, leading to structural maturation of the graft.

Mechanical properties also improved following bioreactor conditioning. Successful grafts typically require suture retention strength >1 N for safe anastomosis; plant scaffolds in these studies achieved 2 N, comparable to native tissues and meeting transplantation standards (Billiar et al., 2001). Burst pressure also increased after conditioning, likely due to enhanced collagen deposition and ECM remodeling. Gorbenko et al. further tested whether high-frequency pulsatile flow (8.75 Hz, simulating a rat heart rate of 525 bpm) provided additional benefits compared to lower-frequency conditioning. The study found that while high-frequency conditioning did not damage the graft, the improvement over standard pulsatile flow was minimal. This finding highlights the need to optimize conditioning parameters to balance mechanical stimulation with cell viability and function.

These results demonstrate that perfusion bioreactor conditioning enhances the biological and mechanical properties of plant-based vascular grafts, preparing them for implantation. Future studies should refine conditioning protocols, optimizing shear stress magnitudes, flow rates, and duration to maximize graft functionality and improve long-term patency *in vivo*.

## Lessons from plant-derived scaffolds for other tissues

While vascular grafts are a primary focus of this review, insights from decellularized plant scaffolds in other tissue engineering applications may inform their development. Researchers have explored the structural properties of plant tissues for regenerating bone, cartilage, muscle, cardiac, and liver tissues, selecting plant structures that mimic target tissue ECM. Understanding how decellularization and functionalization enhance cell adhesion, mechanical properties, and bioactivity could provide strategies to improve vascular graft performance.

Some plant structures, particularly wood and mineral-rich fruits, exhibit high stiffness and porous networks resembling cancellous bone. These examples demonstrate how scaffold stiffness and porosity can be optimized to enhance ECM deposition, critical for vascular graft durability. Lee et al. demonstrated that decellularized apple hypanthium (~300 μm pores) supported osteoblast proliferation and calcium deposition (Lee et al., 2019). Latour et al. further confirmed that apple-derived cellulose scaffolds enhanced osteogenesis, improving mineralization and mechanical stiffness over time (Latour and Pelling, 2022). Tampieri et al. utilized rattan’s natural channels to support biomineralization and osteogenic differentiation, yielding a bone-like material (Tampieri et al., 2009).

In cartilage tissue engineering, the hydrophilic, cellulose- and pectin-rich ECM of plants like cactus and root vegetables supports hydration, essential for cartilage function (Zhang et al., 2022). Cellulose-based hydrogels have demonstrated biocompatibility and mechanical resilience (Svensson et al., 2005; Phatchayawat et al., 2022), suggesting that similar modifications could enhance endothelialization and compliance in vascular grafts.

Plant-derived scaffolds offer fiber alignment that guides myoblast organization for skeletal muscle regeneration. Such structural anisotropy could be adapted to improve SMC alignment and function in vascular grafts. Decellularized spinach leaves preserved venation networks that directed myotube alignment and synchronized contractions under electrical stimulation (Cheng et al., 2020). Decellularized grass blades with parallel fibers supported myotube formation (Allan et al., 2021). Aligned fibers may also serve as channels for nutrient and oxygen delivery, supporting the engineering of contractile muscle tissue.

In cardiac applications, decellularized spinach leaves demonstrated the ability to support neonatal rat cardiomyocyte seeding, promoting synchronized contractions and nutrient transport through the preserved venation network (Gershlak et al., 2017). Plant tissues could serve as templates for cardiac patches, particularly where revascularization is critical. While still in the early stages, this approach highlights the potential of plant-derived scaffolds to support dense, metabolically active cardiac tissue.

Plant-derived scaffolds also show promise for liver tissue engineering. Their structural similarity to liver lobules, high porosity, and biocompatibility make them well-suited for hepatocyte culture. Apple-derived cellulose supported adipose-derived stem cell differentiation into hepatocyte-like cells, expressing key liver markers such as albumin and alpha-fetoprotein (Hu et al., 2024). In a mouse model of acute liver injury, these scaffolds supported liver function recovery by reducing liver injury markers and promoting the formation of vascular and bile duct-like structures. Combining plant scaffolds with 3D bioprinting could expand their application in liver regeneration.

Plant-derived cellulose has gained interest in skin tissue engineering and wound healing due to its moisture-retaining capacity and biocompatibility. These biomaterials enhance skin regeneration by mimicking ECM properties and supporting cellular infiltration (Tarrahi et al., 2022). Advances in functionalizing cellulose wound dressings, including drug-loaded hydrogels and antibacterial modifications, may inform strategies to improve vascular graft endothelialization and host tissue integration. Additionally, the ability of cellulose to regulate hydration and support controlled bioactive molecule release in wound healing could be leveraged to enhance vascular graft remodeling and long-term performance.

The versatility of plant-derived ECM makes it well-suited for various biomedical applications. Leaves, stems, and wood offer scaffolds tailored to specific needs, while chemical modifications further expand their utility. Mineralization can enhance rigidity for bone regeneration, while gelatin coatings can improve cell adhesion for soft tissues. Plant-derived cellulose can also be functionalized with RGD peptides to enhance adhesion or heparin to confer anticoagulant properties (Yun et al., 2023). Although many applications are in early stages, plant-based scaffolds represent a shift toward scalable, customizable biomaterials. As research progresses, decellularized plant-derived scaffolds may advance into preclinical trials, offering a low-cost, sustainable alternative to traditional synthetic and animal-derived biomaterials.

While plant-derived scaffolds exhibit versatility across tissue engineering applications, their most immediate clinical impact may be in vascular grafts, where their scalability and biocompatibility address a significant clinical need. Strategies developed for optimizing plant-based biomaterials in other tissues, such as structural reinforcement, bioactive coatings, and controlled degradation, can guide further refinements in vascular graft technology. The success of plant scaffolds in supporting mineralization (bone) and contractile tissues (muscle/heart) suggests that mechanical reinforcement and anisotropy are key approaches that can be applied to designing more robust vascular grafts.

## Challenges and future directions

Despite significant progress, several challenges must be addressed before decellularized plant vascular grafts can transition to clinical applications. Ensuring consistent scaffold properties across production batches requires standardization of decellularization protocols, immunogenicity assessment, biodegradability control, and rigorous in vivo testing.

### Standardization of decellularization

Decellularization methods vary widely, leading to inconsistent scaffold composition and mechanical integrity. Standardized, reproducible protocols are essential for scalable production and clinical translation. Detergent concentration, exposure time, and enzymatic digestion must be optimized to achieve complete cell removal while preserving scaffold integrity. A DNA threshold (<50 ng/mg) could serve as a benchmark for quality control, ensuring sufficient removal of plant cellular material.

Advanced monitoring, including optical imaging and biochemical assays, could enable real-time quality control, similar to approaches used for animal-derived scaffolds (Merna et al., 2013). Automated perfusion systems could improve reproducibility by maintaining uniform exposure to detergents and rinsing solutions. Additionally, reducing residual plant biomolecules is critical to prevent immune reactions (Fontana et al., 2017).

Standardizing mechanical testing is equally important. Variability in burst pressure, compliance, and suture retention methods complicate comparisons across studies. Establishing standardized testing protocols aligned with ASTM or ISO guidelines would facilitate accurate benchmarking.

Addressing these standardization challenges will be essential for transitioning plant-derived scaffolds from experimental models to clinically viable biomaterials. Future research should focus on optimizing decellularization efficiency, refining mechanical characterization protocols, ensuring batch-to-batch reproducibility, and developing scalable production methods suitable for regulatory approval and clinical use.

### Removal of immunogenic components

While cellulose is largely biocompatible, residual proteins, DNA, and endotoxins could elicit immune responses if not fully removed. Residual plant material can trigger transient inflammation (Modulevsky et al., 2016). Decellularization protocols should incorporate digestion with proteases and extensive rinsing to eliminate cellular debris. Optimizing these steps is critical for producing immunologically inert scaffolds suitable for medical applications.

### Biodegradability

A key consideration for plant-derived scaffolds is their biodegradability, as mammals lack enzymes to degrade cellulose. Decellularized plant scaffolds could persist in vivo for extended periods, functioning as permanent implants. Long-term stability may support tissue remodeling but risks chronic inflammation or fibrotic encapsulation. Mouse studies show that cellulose induces mild inflammation, which diminishes over eight weeks (Modulevsky et al., 2016).

Future strategies could modify biodegradability by incorporating degradative enzymes, promoting oxidative breakdown, or coating the scaffold with bioresorbable materials. Further studies on cellulose-host interactions are needed to optimize scaffold replacement by native ECM and balance mechanical stability with biodegradability for long-term graft function.

### Cell sourcing and endothelialization strategy

Scalable cell seeding is essential for clinical use. Autologous cell seeding improves graft patency but is costly and time-intensive. Alternatives include immune-evasive allogeneic cells or functionalized grafts that recruit host endothelial progenitor cells using VEGF, heparin, or anti-CD34 antibody immobilization. Nitric oxide-releasing coatings and extracellular vesicles are also promising. Modifying scaffold surfaces to accelerate host cell recruitment could enable rapid, cell-free endothelialization, as demonstrated in synthetic and animal-derived grafts.

Given that the long-term success of vascular grafts depends on stable endothelial coverage and functional SMC integration, future research should explore whether plant-derived scaffolds can be optimized for rapid, cell-free endothelialization. Studies on synthetic grafts and decellularized animal scaffolds have demonstrated that modifying surface chemistry or incorporating bioactive factors can significantly accelerate host cell recruitment. Investigating whether similar strategies can be applied to plant-based grafts will be crucial for their clinical viability.

### Long-term in vivo performance

Most research on plant-derived vascular grafts has been conducted in vitro and advancing to *in vivo* models is a crucial next step. Key questions remain: Can plant-based grafts maintain patency and function under arterial circulation? Will the endothelial layer remain stable to prevent thrombosis? How will immune cells respond, will macrophages attempt to degrade the cellulose scaffold, or will it remain inert? Will the graft exhibit appropriate mechanical responses, such as dilation and contraction with blood pressure, or will compliance mismatch lead to complications?

Short-term studies, such as CAM assays and peripheral implants in rodents, suggest good biocompatibility with minimal acute rejection. However, arterial implantation in rat or rabbit models is essential to assess patency, immune response, and remodeling under physiological flow over longer timeframes. Calcification remains a major concern, as mineral deposition could stiffen the scaffold, reduce compliance, and lead to failure. Monitoring patency and calcification using Doppler ultrasound or CT, along with histological analysis of explanted grafts, will be necessary to assess cell retention, fibrosis, and calcification. Another challenge is ensuring that thicker plant scaffolds receive sufficient nutrient and oxygen diffusion to support cell viability.

Encouragingly, plant-derived scaffolds have demonstrated favorable biocompatibility in non-vascular applications. In one study, decellularized apple scaffold implanted into a rat skull defect integrated with host tissue without immune rejection (Lee et al., 2019). While this suggests that systemic toxicity may not be a significant concern, vascular grafts face greater mechanical and hemodynamic stresses. Large-animal studies, such as pig models, will be necessary to evaluate long-term graft durability, remodeling potential, and suitability for clinical translation.

### Regulatory considerations

Regulatory approval will require detailed characterization of scaffold composition, mechanical performance, sterility, and biocompatibility. FDA-approved cellulose hemostats like Surgicel, ActCel, and Tabotamp demonstrate feasibility, and these precedents may ease acceptance of vascular grafts (Levy, 2013). The absence of animal components reduces zoonotic risks, a significant challenge for animal-derived grafts. Bioengineers, material scientists, and surgeons must work closely to refine graft properties, while industry partners must develop GMP-compliant production.

Sterilization remains challenging. Ethylene oxide and gamma irradiation can compromise cellulose structure. Supercritical CO_2_ treatment offers a promising alternative, combining decellularization and sterilization without damaging the scaffold (Harris et al., 2021). Identifying an effective and scalable sterilization approach will be essential for regulatory approval and clinical adoption.

### Public perception and continued innovation

Plant scaffolds avoid ethical concerns associated with animal and human-derived biomaterials, which may enhance public acceptance (Duarte et al., 2023). Still, plant-derived materials remain unconventional, and patient confidence will depend on clear communication regarding safety and efficacy. Generating strong clinical data will be critical in establishing credibility and regulatory approval.

Future research may explore genetically engineered plants optimized for tissue engineering, such as reducing lignin or modifying vascular patterns, and integrating 3D bioprinting with plant-derived ECM. Functionalization with collagen, elastin, or heparin can further improve endothelialization, mechanical resilience, and biocompatibility.

If challenges in standardization, immunogenicity, and validation are overcome, plant-derived vascular grafts could offer scalable, cost-effective alternatives for treating cardiovascular disease. With healthcare costs for cardiovascular disease continuing to rise (Tsao et al., 2023), the ability to produce grafts sustainably may redefine regenerative medicine and expand access to life-saving, off-the-shelf technologies.

## Conclusions

Decellularized plant scaffolds offer a sustainable, scalable alternative to synthetic and animal-derived biomaterials. Their natural vascular architecture, biocompatibility, and versatility make them strong candidates for small-caliber vascular grafts. Recellularization with endothelial and SMCs, combined with bioreactor conditioning, enhances endothelial alignment, reduces thrombogenicity, and promotes smooth muscle maturation, creating grafts that more closely mimic native vessels. Plant-derived scaffolds are also being explored for bone, cartilage, skeletal muscle, cardiac, and liver tissue engineering, showcasing their versatility in regenerative medicine. Clinical translation will require standardizing decellularization, ensuring immunological safety, refining biodegradability, optimizing scaffold functionalization, and validating performance in large-animal models. If these hurdles are addressed, plant-derived scaffolds could offer cost-effective, off-the-shelf solutions for cardiovascular disease and other tissue engineering challenges. These bioengineered grafts may provide a paradigm shift in medical biomaterials, merging biotechnology with plant science to create scalable, ethical solutions for unmet clinical needs. Decellularized plant scaffolds could play an integral role in next-generation tissue engineering by repurposing nature’s vascular structures for human health.

## Figures and Tables

**Table 1.** Comparison of vascular graft types. Autologous, synthetic, decellularized animal, and plant-derived grafts differ in availability, thrombogenicity, compliance, remodeling, immune response, biodegradability, and clinical use. While autologous grafts are ideal, their limited supply drives interest in synthetic and bioengineered alternatives.

Material	Autograft (Saphenous Vein, IMA)	Synthetic (ePTFE, Dacron)	Decellularized Animal (SIS, Pericardium, UAA)	Decellularized Plant (Leatherleaf, Parsley)
Availability	Limited, patient-dependent	Unlimited, off-the-shelf	Moderate, donor-dependent	High, scalable production
Thrombogenicity	Low, native endothelium	High, unless endothelialized	Moderate, requires endothelialization	Moderate, requires endothelialization
Compliance	Matches native artery	Mismatch, stiffer than native vessels	Varies, can be engineered to match native artery	Varies, can be engineered to match native artery
Long-Term Remodeling	Excellent, integrates with host tissue	Poor, non-degradable, no remodeling	Moderate, host cell infiltration occurs	Unknown, host remodeling potential under investigation
Immune Response	None, self-derived	Low, non-immunogenic polymers	Variable, residual antigens/DNA trigger immune response	Low, lacks species-specific antigens
Risk of Disease Transmission	None	None	Potential, zoonotic pathogens must be removed	None, no animal-derived components
Biodegradability	Yes, replaced by host ECM	No, remains permanently	Partial, varies by material	No, cellulose is non-degradable
Permeability	Low, non-porous	Highly variable	Moderate, can be engineered	Minimal leakage
Standardization & Variability	Uniform properties, patient-specific	Highly controlled	Moderate, depends on decellularization process	Varies by leaf collection site
Clinical Use	Gold standard	Common for large grafts	Emerging, limited approval	Experimental, preclinical stage

IMA: internal mammary artery; SI: small intestinal submucosa; UAA: umbilical artery allograft.
